# Schizandrin Protects against OGD/R-Induced Neuronal Injury by Suppressing Autophagy: Involvement of the AMPK/mTOR Pathway

**DOI:** 10.3390/molecules24193624

**Published:** 2019-10-08

**Authors:** Guangyun Wang, Tiezheng Wang, Yuanyuan Zhang, Fang Li, Boyang Yu, Junping Kou

**Affiliations:** 1 State Key Laboratory of Natural Products, Jiangsu Key Laboratory of TCM Evaluation and Translational Research, Department of Pharmacology of Chinese Material Medica, School of Traditional Chinese Pharmacy, China Pharmaceutical University, Nanjing 211198, China; wgyhefei@163.com (G.W.); 18168020636@163.com (T.W.); cpuzhang27@163.com (Y.Z.); lifang19870801@126.com (F.L.); 2 State Key Laboratory of Natural Products, Jiangsu Key Laboratory of TCM Evaluation and Translational Research, Department of Resource and Developmemt of Chinese Material Medica, School of Traditional Chinese Pharmacy, China Pharmaceutical University, Nanjing 211198, China; boyangyu59@163.com

**Keywords:** OGD/R, autophagy, AMPK/mTOR, schizandrin, ischemic stroke

## Abstract

The neuroprotective role of schizandrin (SA) in cerebral ischemia-reperfusion (I/R) was recently highlighted. However, whether SA plays a regulatory role on autophagy in cerebral I/R injury is still unclear. This study aimed to explore whether the neuroprotective mechanisms of SA were linked to its regulation of AMP-activated protein kinase (AMPK)/mammalian target of rapamycin (mTOR)/autophagy pathway in vivo and in vitro. The present study confirmed that SA significantly improved oxygen-glucose deprivation/re-oxygenation (OGD/R)-induced PC12 cells injury. The results of immunoblotting and confocal microscope showed that SA decreased autophagy in OGD/R-injured PC12 cells, which was reflected by the decreased Beclin-1 and LC3-II expression, autophagy flux level, and LC3 puncta formation. In addition, the autophagy inducer rapamycin partially prevented the effects of SA on cell viability and autophagy after OGD/R, whereas the autophagy inhibitor 3-methyladenine (3-MA) exerted the opposite effect. The results of Western blotting showed that SA markedly decreased the phosphorylation of AMPK (p-AMPK), whereas the phosphor-mTOR (p-mTOR) levels increased in the presence of OGD/R insult. Furthermore, pretreatment with the AMPK inducer AICAR partially reversed the protective effects and autophagy inhibition of SA. However, AMPK inhibitor Compound C pretreatment further promoted the inhibition of SA on autophagy induction and cell damage induced by OGD/R. Taken together, these findings demonstrate that SA protects against OGD/R insult by inhibiting autophagy through the regulation of the AMPK-mTOR pathway and that SA may have therapeutic value for protecting neurons from cerebral ischemia.

## 1. Introduction

Ischemic stroke, also known as cerebrovascular accident, is characterized by an insufficient oxygen supply and restoration of blood flow [1]. It is a frightening anomaly that kills millions of people all over the world every year and, in addition to a high mortality rate, also causes a high degree of disability. Stroke has a significant global impact; currently, the only Food and Drug Administration (FDA)-approved pharmacotherapy for acute stroke includes intravenous thrombolytic treatment with a recombinant tissue plasminogen activator (rtPA). This strategy has a short therapeutic window and a risk for intracerebral hemorrhage, making it safe and effective only in a subset of patients [2,3,4]. Therefore, cutting-edge research for novel targets and drugs to manage stroke is strongly needed.

Autophagy is an evolutionarily conserved process for the bulk degradation and recycling of cytosolic proteins and organelles [5]. As a degradation/recirculation system, autophagy is believed to play an important role in pathological conditions in many organs, including cerebral ischemia [6,7]. Many studies have found that neuronal death is tightly associated with autophagy. Consequently, autophagy in neurons upon ischemic insult was believed to be a type of cell death and exhibited detrimental effects in ischemic stroke. Previous studies have shown that autophagy is one of several morphological features that occur during cell death after brain ischemia, and the inhibition of autophagy with a selective autophagy inhibitor 3-methyladenine (3-MA) exerts protective effects [8]. Thus, suppression of excessive neuronal autophagy was considered for preventing ischemic stroke.

Adenosine monophosphate-activated protein kinase (AMPK), a member of the serine/threonine (Ser/Thr) kinase group, was shown to initiate autophagy. The mammalian target of rapamycin (mTOR) is a conserved serine/threonine kinase that regulates cell growth and autophagy. AMPK is an upstream regulator of mTOR in mammalian cells, and it is well-known that autophagy is promoted by AMPK and inhibited by mTOR [9]. Accumulating evidence demonstrates that the AMPK/mTOR signaling pathway can modulate autophagic activation following cerebral ischemia-reperfusion (I/R) [10]. Therefore, the AMPK/mTOR signaling pathway may be a novel therapeutic target for treating cerebral I/R injury by modulating autophagy.

Schizandrin A (SA) is a bioactive lignin compound isolated from *Schisandra chinensis* [11]. It shows several cytoprotective activities, including anticancer [12], anti-inflammatory [13], and anti-liver-injury activities [14]. In addition, it was reported that SA can improve cerebral ischemia reperfusion injury to exert neuroprotective effect [15,16]. SA prevents oxygen-glucose deprivation/re-oxygenation (OGD/R)-induced cell death in primary cortical neurons through the JNK and ERK pathway [15]. SA also protects against cerebral I/R injury by suppressing inflammation and oxidative stress, and this effect is regulated by the AMPK/Nrf2 pathway [16]. Moreover, our previous studies showed that SMXZF (a combination of Rb1, Rg1, schizandrin, and DT-13 (6:9:5:4) derived from Sheng-Mai San) displays neuroprotective effects against I/R injury, which is associated with autophagy inactivation through the AMPK/mTOR and JNK pathways [17]. However, there is no direct evidence that SA can play a neuroprotective role by inhibiting ischemia-induced neuronal autophagy through the AMPK/mTOR pathway. Therefore, in the present study, we investigated the effects of SA against OGD/R-induced autophagy and the AMPK/mTOR pathways in PC12 cells. These findings are expected to provide further evidence for the application of SA in cerebral diseases such as stroke.

## 2. Results

### 2.1. SA Protects Cells against OGD/R Injury in PC12 Cells

**Figure 1:**
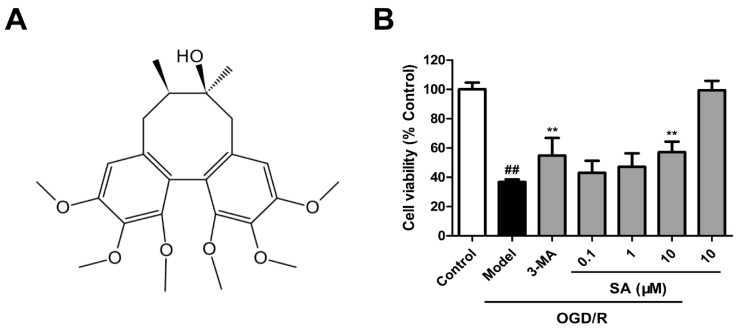
Schizandrin A (SA) protects cells against OGD/R injury in PC12 cells. (**A**) The chemical structure of ginsenoside GRb1. (**B**) Cell viability was determined using the MTT assay (n = 6). All data are mean ± SD. ^##^ *P* < 0.01 vs. Control group; ** *P* < 0.01 vs. OGD/R group.

An OGD/R model was utilized with differentiated PC12 cells to further investigate the neuroprotective effects of SA (chemical structure shown as Figure 1A) in vitro. The MTT assay (Figure 1B) showed that OGD/R induced a significant decrease in cell viability compared to control cells, whereas the SA (10 μM) and 3-MA treatment significantly increased cell survival, respectively. SA (10 μM) significantly reversed cell death caused by OGD/R injury.

### 2.2. SA Inhibits Autophagy Following OGD/R in PC12 Cells

**Figure 2:**
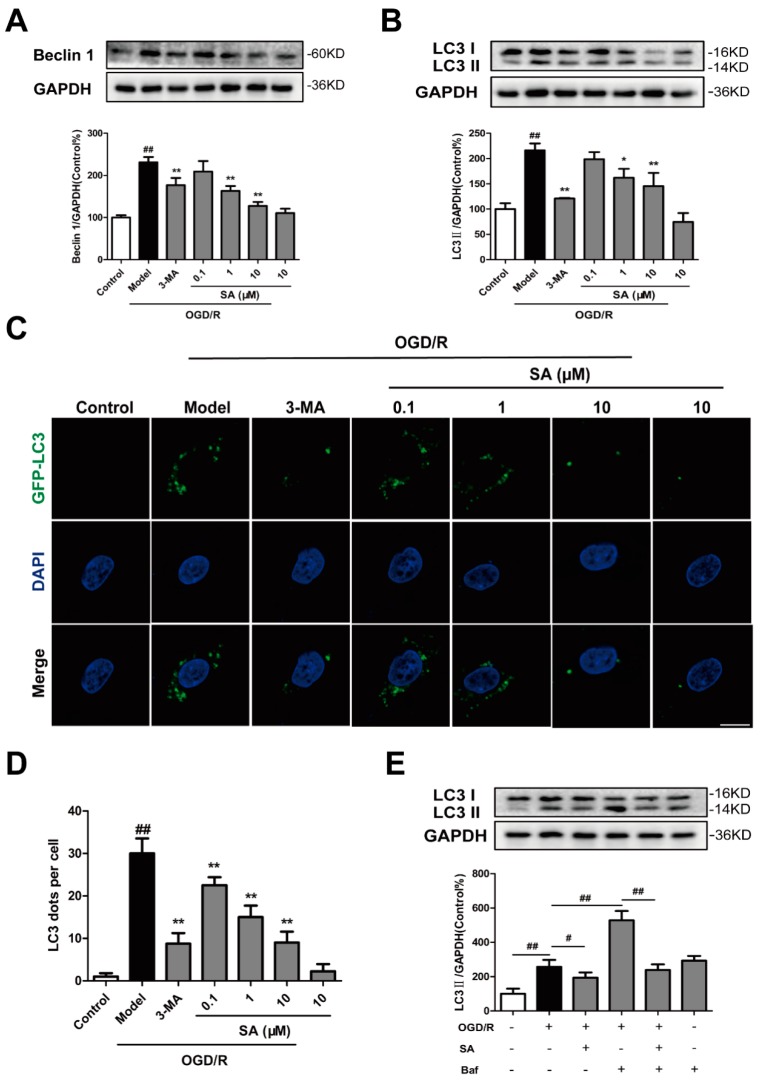
SA inhibits autophagy following OGD/R in PC12 cells. PC12 cells were pretreated with SA and exposed to OGD/R. The expression of Beclin 1 (**A**) and LC3 (**B**) was detected by Western blot (n = 3). (**C**) LC3-positive puncta in cells were detected by GFP-LC3 plasmid transfection. Scale bar: 10 μm. (**D**) The number of LC3 puncta in PC12 cells were calculated (n = 4). ^##^ *P* < 0.01 vs. control group; * *P* < 0.05 and ** *P* < 0.01 vs. model group. (**E**) PC12 cells were treated with Baf combined with SA, and cell lysates were prepared for analyzing the LC3 expression (n = 3). ^#^ *P* < 0.05 and ^##^ *P* < 0.01.

Next, we tested whether SA regulates autophagy in our in vitro ischemic model. Beclin1 and LC3 are biomarkers for autophagy activation in mammalian cells. To examine autophagic activity after OGD/R and to determine whether SA could inhibit the activation of autophagy, we tested the protein expression of LC3 and Beclin1 in the ischemic PC12 cells. We found that the level of Beclin1 and LC3-II following OGD/R was significantly greater than that following the control treatment and that SA (1 and 10 μM) could significantly inhibit the increased expression of Beclin1 (Figure 2A) and LC3-II (Figure 2B) following OGD/R. The number of LC3-positive puncta per cell was determined by LC3 plasmid transfection. As shown in Figure 2C,D, either SA (0.1–10 μM) or 3-MA pretreatment effectively inhibited the OGD/R-induced increase of LC3 dots in PC12 cells. However, compared with the control group, 3-MA and SA did not significantly reduce the level of autophagy (Figure 2B and Figure S1A). In addition, we also examined the effect of SA on autophagic flux. We found that SA treatment resulted in decreased LC3-II levels in PC12 cells following Bafilomycin A1 (Baf) treatment, compared with the model group treated with Baf (Figure 2E). It suggested that SA could inhibit the autophagosome formation at the early stage of autophagy. Interestingly, the level of LC3-II following Baf treatment was significantly greater than that of the control group, while SA treatment did not inhibit the Baf-induced increase of LC3-II levels in PC12 cells (Figure S1B).

### 2.3. Autophagy Contributes to the Neuroprotective Effects of SA in PC12 Cells

**Figure 3:**
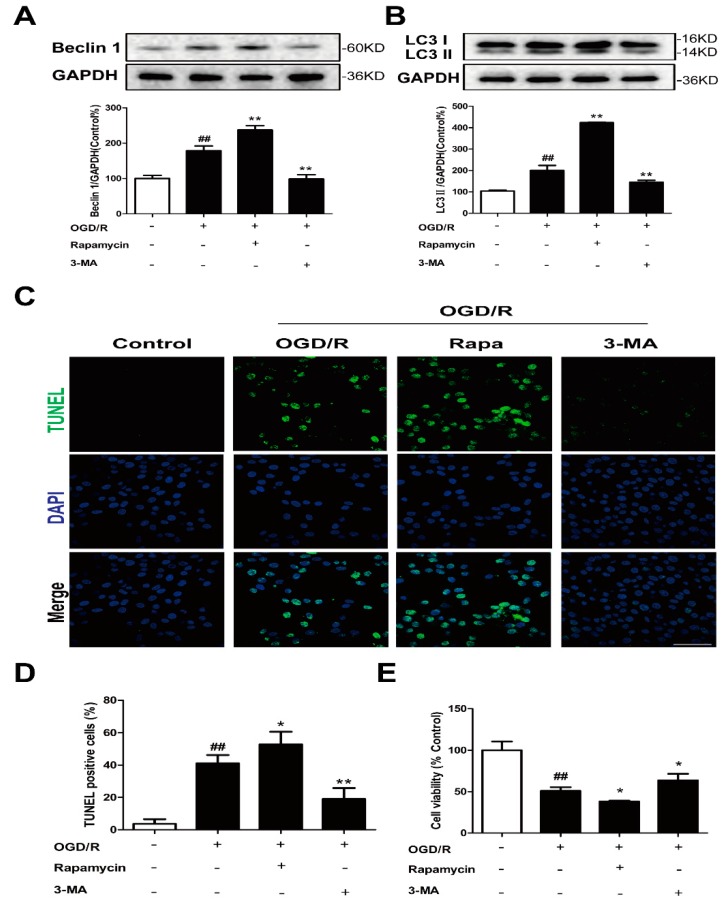
Autophagy is a mechanism for OGD/R-induced cell death in PC12 cells. PC12 cells were treated with 0.1 μM rapamycin or 3 mM 3-MA and then exposed to OGD/R. The expression of Beclin 1 (**A**) and LC3 (**B**) was detected by Western blot (n = 3). (**C**,**D**) Apoptotic cell death was detected by TUNEL staining (n = 6). Scale bar: 20 μm. (**E**) Cell viability was determined using the MTT assay (n = 6). All data are mean ± SD. ^##^ *P* < 0.01 vs. control group; * *P* < 0.05 and ** *P* < 0.01 vs. model group.

PC12 cells were treated with the autophagy inducer rapamycin and inhibitor 3-MA to determine the role of autophagy in our in vitro ischemia model. The results of the Western blotting showed that the increased Beclin 1 (Figure 3A) and conversion of LC3-I to LC3-II (Figure 3B) induced by OGD/R were further enhanced by rapamycin treatment. Rapamycin can also significantly enhance basal autophagy in PC12 cells (Figure S1A). Treatment with rapamycin significantly increased the number of TUNEL positive cells (Figure 3C,D) and decreased cell viability (Figure 3E) after OGD/R. However, 3-MA treatment significantly antagonized OGD/R-triggered autophagy induction and cell injury (Figure 3). Therefore, autophagy is a mechanism for OGD/R-induced cell death.

**Figure 4:**
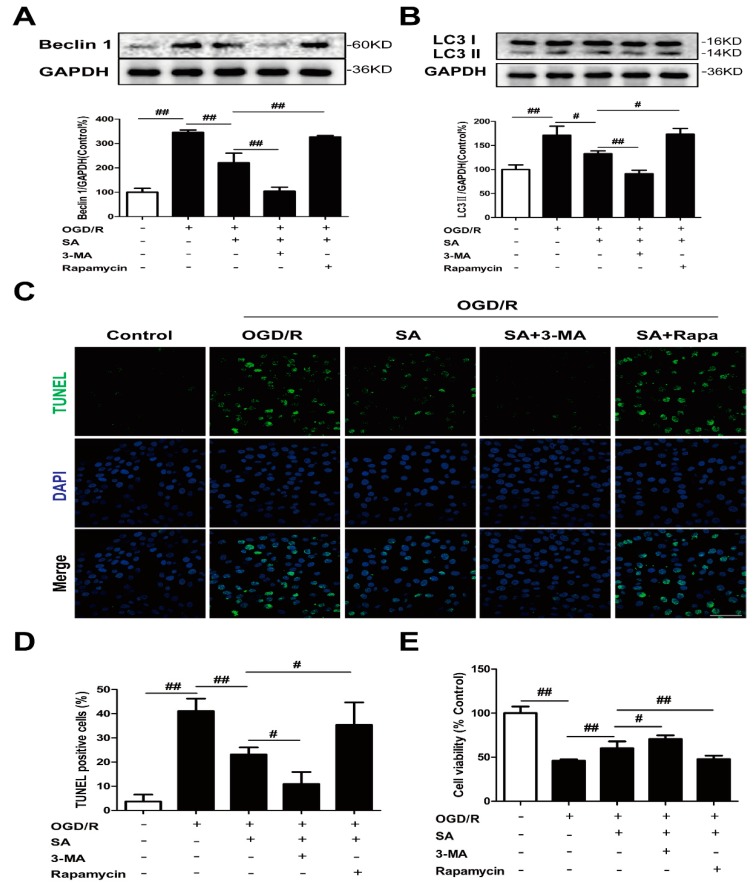
Autophagy contributes to the neuroprotective effects of SA in PC12 cells. PC12 cells were treated with SA (10 μM) and 0.1 μM rapamycin or 3 mM 3-MA and then exposed to OGD/R. The expression of Beclin 1 (**A**) and LC3 (**B**) was detected by Western blot (n = 3). (**C**,**D**) Apoptotic cell death was detected by TUNEL staining (n = 6). Scale bar: 20 μm. (**E**) Cell viability was determined using the MTT assay (n = 6). All data are mean ± SD. ^#^ *P* < 0.05 and ^##^ *P* < 0.01.

To determine whether autophagy was involved in SA-induced protection, PC12 cells were co-incubated with SA and autophagy inhibitor 3-MA or autophagy agonist rapamycin. Treatment with SA not only obviously reduced the levels of Beclin-1 (Figure 4A) and LC3 II (Figure 4B), but also improved TUNEL positive cells (Figure 4C,D) and cell viability (Figure 4E), which were blocked by treatment with rapamycin after OGD/R. On the contrary, 3-MA treatment further promoted the inhibition of SA on autophagy induction and cell damage induced by OGD/R (Figure 4).

### 2.4. Involvement of the AMPK-mTOR/Autophagy Pathway in SA-Induced Protection in PC12 Cells

**Figure 5:**
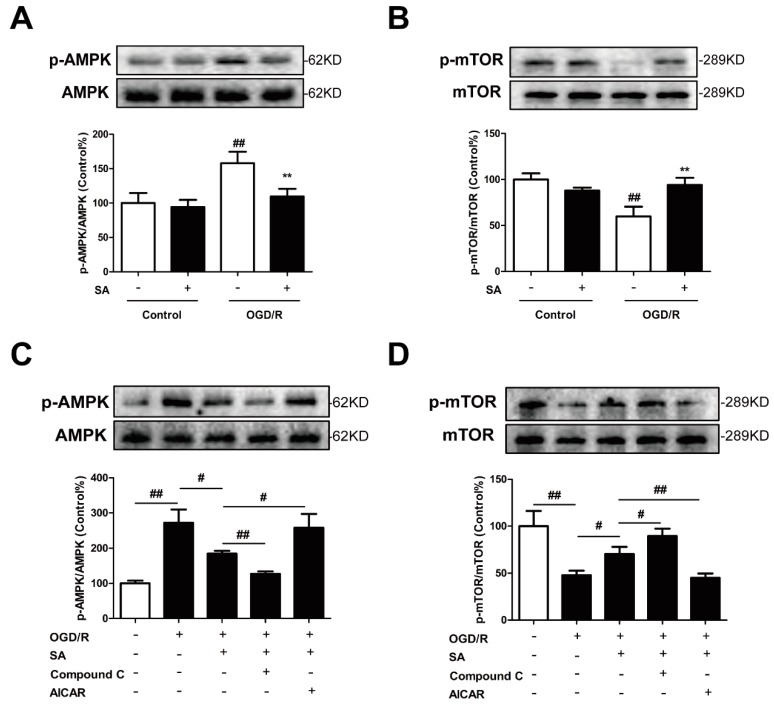
SA modulated AMPK/mTOR pathway proteins in PC12 cells. PC12 cells were pretreated with SA and exposed to OGD/R. The expression of phosphorylation of AMPK (**A**) and mTOR (**B**) was detected by Western blot (n = 3). All data are mean ± SD. ^##^ *P* < 0.01 vs. control group; ** *P* < 0.01 vs. model group. PC12 cells were treated with SA (10 μM) and 1 μM compound C or 1 mM AICAR and then exposed to OGD/R. The expression of phosphorylation of AMPK (**C**) and mTOR (**D**) was detected by Western blot (n = 3). All data are mean ± SD. ^#^ *P* < 0.05 and ^##^ *P* < 0.01.

We then investigated whether SA could regulate autophagy by affecting the AMPK/mTOR pathway in PC12 cells. As indicated in Figure 5A, SA significantly decreased the OGD/R-induced increase in protein expression of phosphorylation level of AMPK. In contrast, SA obviously increased the OGD/R-mediated decrease in the phosphorylation level of mTOR (Figure 5B). Of note, activation of the AMPK pathway by the AMPK inducer AICAR significantly abrogated the SA-mediated decrease in the p-AMPK/AMPK ratio and the increase in the p-mTOR/mTOR ratio (Figure 5C,D). On the contrary, the AMPK inhibitor Compound C pretreatment further promoted the effects of SA on the p-AMPK and p-mTOR expression induced by OGD/R (Figure 5C,D).

**Figure 6:**
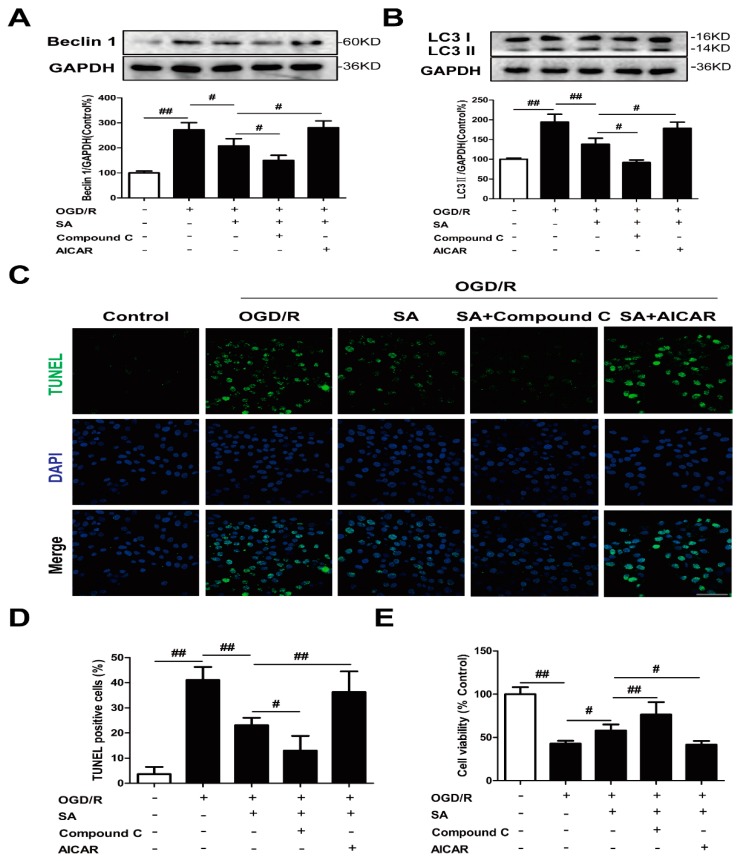
Involvement of the AMPK-mTOR/autophagy pathway in SA-induced protection. PC12 cells were treated with SA (10 μM) and 1 μM Compound C or 1 mM AICAR and then exposed to OGD/R. The expression of Beclin 1 (**A**) and LC3 (**B**) was detected by Western blot (n = 3). (**C**,**D**) Apoptotic cell death was detected by TUNEL staining (n = 6). Scale bar: 20 μm. (**E**) Cell viability was determined using the MTT assay (n = 6). All data are mean ± SD. ^#^ *P* < 0.05 and ^##^ *P* < 0.01.

Subsequently, we investigated the effects of the activation of the AMPK/mTOR pathway on the SA-mediated suppression of autophagy under OGD/R. Data revealed that activation of the AMPK/mTOR pathway by AICAR treatment significantly abolished the SA-mediated autophagy suppression, as evidenced by an increase in Beclin 1 (Figure 6A) and LC3-II (Figure 6B) protein expression plus TUNEL positive cells (Figure 6C,D) and a decrease in cell viability (Figure 6E) compared with the SA alone-treated group. Simultaneously, Compound C pretreatment further promoted the inhibition of SA on autophagy induction and cell damage induced by OGD/R (Figure 6). Collectively, these results indicated that AMPK/mTOR signaling was involved in the SA-mediated suppression of autophagy and subsequent neuroprotective effect in OGD/R-exposed PC12 cells. Interestingly, the results of the Western blotting showed that the increased conversions of LC3-I to LC3-II induced by OGD/R were further enhanced by AICAR treatment. However, Compound C treatment significantly antagonized OGD/R-triggered autophagy (Figure S1C).

### 2.5. SA Inhibits AMPK/mTOR Pathway and Autophagy Following Cerebral Ischemia/Reperfusion Injury in Mice

**Figure 7:**
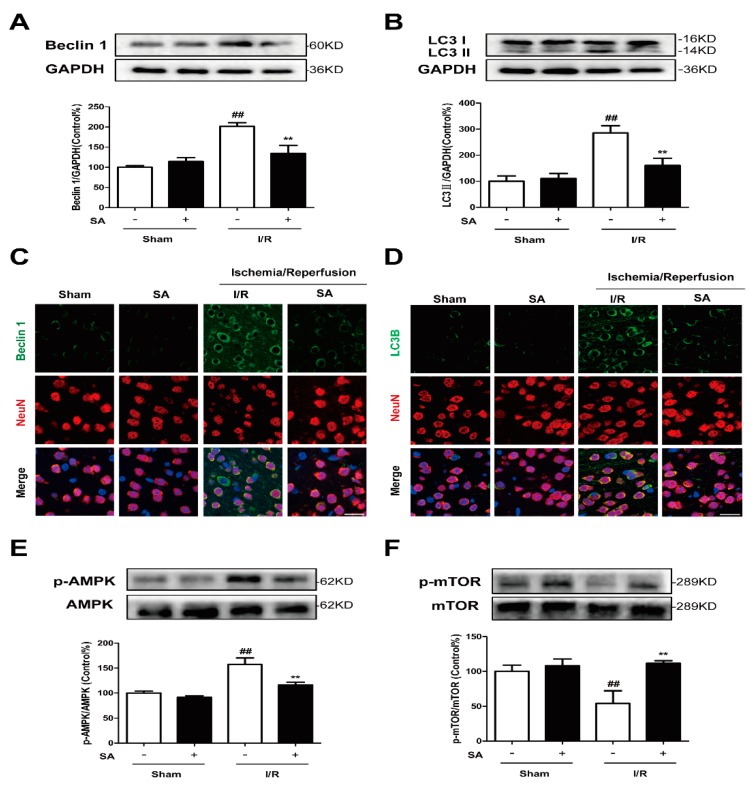
SA Inhibits AMPK/mTOR Pathway and Autophagy Following Cerebral Ischemia/Reperfusion Injury in Mice. Mice were pretreated with SA and exposed to I/R. (**A**,**B**) The expression of Beclin 1 (**A**) and LC3 (**B**) was detected by Western blot (n = 3). (**C**,**D**) Then confocal microscope was used to detect Beclin 1/LC3B (green) and Neun (red). Bar: 20 µm. (**E**,**F**) The expression of phosphorylation of AMPK (E) and mTOR (F) was detected by Western blot (n = 3). All data are mean ± SD. ^##^ *P* < 0.01 vs. Sham group; ** *P* < 0.01 vs. I/R group.

Previous studies showed that SA treatment significantly improved the neurological score and reduced infarct volume 24 h after reperfusion in a in vivo MCAO model [15]. In our experiments, HE staining also confirmed that SA can significantly improve cerebral ischemia-reperfusion injury in mice, which is consistent with previous studies (Figure S1D). To investigate the possible mechanism of the SA-mediated neuroprotective effect, we further evaluated its effects on the autophagy and AMPK/mTOR pathway. We found that the level of Beclin1 and LC3-II following I/R were significantly greater than that following the sham treatment and that SA could significantly inhibit the increased expression of Beclin1 and LC3-II following I/R (Figure 7A,B). Simultaneously, GRb1 could also significantly reduce the increase of Beclin 1 (Figure 7C) and LC3B (Figure 7D) fluorescence intensity in neurons caused by I/R. As shown in Figure 7E and F, compared with the levels in the sham group, the level of phosphorylated AMPK was markedly increased, while the level of phosphorylated mTOR was significantly decreased following I/R. After SA treatment, both of them tended to return to normal: the level of phosphorylated AMPK was decreased and phosphorylated mTOR was increased (Figure 7E,F).

## 3. Discussion

In present study, we used PC12 cells to examine the regulatory effect of SA on autophagy and its significance in cell injury induced by OGD/R, because PC12 cells are a powerful in vitro model system for deciphering the molecular events that confer the hypoxia-resistant and oxygen-sensing phenotypes [18]. Moreover, it was confirmed that OGD/R can activate autophagy in PC12 cells [19,20,21], which provides an important platform for researchers to study the molecular mechanism of autophagy-related central nervous system diseases and the intervention of drugs. Therefore, we used the OGD/R model of PC12 cells to simulate cerebral ischemia in vitro.

In the previous literatures, several studies reported that SA exhibited a neuroprotective effect in different cell lines, such as BV-2 cells and primary microglia cells [22], SH-SY5Y cells [23], and rat cortical cells [15,24]. In the glutamate-induced toxicity in rat cortical cells, SA effectively inhibited the increase of intracellular calcium ions (Ca^2+^) and significantly improved the glutathione defense system, as well as inhibited the formation of cellular peroxide [24]. SA played a major role in microglia-mediated neuroinflammation by the TRAF6-NF-κB and Jak2-Stat3 signaling pathways [22]. SA was also reported to be effective in ischemic stroke. SA was exhibited to play an anti-apoptotic activity by modulating the ERK, JNK, and caspase-3 expressions in OGD/R-induced rat cortical cells [15]. Further, SA protects against cerebral I/R injury in vivo and in vitro by suppressing inflammation and oxidative stress, and this effect is regulated by the AMPK/Nrf2 pathway [16]. In this study, we found that SA (10 μM) significantly reversed cell death caused by OGD/R injury (Figure 1), which was consistent with previous reports. Although SA showed neuroprotective activity by regulating various signaling pathways, whether SA exerts neuroprotective effects through autophagy and the related mechanisms remain unclear.

Autophagy is a catabolic process in which worn-out proteins and organelles are destroyed in cells in a lysosome-dependent pathway in order to restore the homeostatic balance [25]. It was proved that an aberrant autophagy process can cause many diseases [26]. In a normal physiological state, autophagy plays a role in regulating cellular homeostatic functions, but in the case of disease, autophagy is activated and plays both defensive and pernicious roles [27]. In ischemic brain injury, autophagy is a double-edged sword and has controversial functions [28]. Increasing evidence suggests that increased autophagy acts as a detrimental mechanism in I/R injury [29,30,31,32,33]. Therefore, the modulation of autophagy could potentially aid in the prevention or the treatment of ischemic stroke. In our in vitro model, moderate protection, as demonstrated by the decreased number of TUNEL positive cells (Figure 3C,D) and increased cell viability (Figure 3E), was found in 3-MA-treated PC12 cells after OGD/R exposure, which was consistent with previous data that showed that autophagy was a mechanism for OGD/R-induced cell death.

To date, researchers have developed many methods to measure autophagy in cells and in animals [34]. Beclin1 and LC3 are key proteins for autophagy. In the early stages of autophagy, Beclin1 (Atg6) can promote the nucleation of the autophagic vesicle and recruit proteins from the cytosol [17]. LC3 (Atg8) is modified to LC3 I, and LC3-I is further converted to an autophagosome associating form, LC3II. LC3-II is stably associated with the autophagosome membrane, and its detection is widely used to measure cellular autophagy [35]. Additionally, the detection of autophagic flux is also an important method to evaluate the degree of autophagic activation. Autophagic flux refers to the entire process of autophagy, which encompasses the inclusion (or exclusion) of cargo within the autophagosome, the delivery of cargo to lysosomes, and its subsequent breakdown and release of the resulting macromolecules back into the cytosol [34]. Autophagic flux is often inferred on the basis of the LC3-II turnover, measured by Western blot in the presence and absence of lysosomal degradation. Lysosomal degradation can be prevented through the use of compounds that neutralize the lysosomal pH, such as Baf A1 and chloroquine. Moreover, GFP-LC3 puncta can also be used to monitor the autophagic flux, because constant increase in the number of cells accumulating GFP-LC3 puncta is suggestive of defective fusion of autophagosomes with lysosomes [34].

Several publications revealed the regulation of schisandrins on autophagy. Schisandrin A improved D-galactosamine-induced acute liver injury through activation of autophagy [36]. Schisandrin B could play a protective role through suppression of autophagy in cyclosporine A induced nephrotoxicity [37] and Aβ-infused rats [38]. The inducing effects of Schisandrin B on cell autophagy may contribute to its liver toxic effects [39]. Schisandrin C enhanced the regulation of autophagy to exert an anti-oxidative mechanism [40] or promote odontoblastic differentiation of human dental pulp cells [41]. For the conflicting roles of schisandrins on autophagy between different investigations, several reasons are considered, such as different cell types and drug treatment time. In our experiment, the levels of Beclin1 (Figure 2A) and LC3 II (Figure 2B) were significantly increased in the OGD/R group, and SA could decrease the expression of Beclin1 and LC3 II. Moreover, SA pretreatment effectively inhibited OGD/R-induced increase of LC3 dots in PC12 cells (Figure 2C,D). In addition, SA-treatment resulted in decreased LC3-II levels following Baf treatment, compared with the model group treated with Baf (Figure 2E). In conclusion, our results strongly indicated that SA protected against OGD/R-induced neuronal injury by inhibiting autophagy in vivo and in vitro, which was also confirmed by the reversed effects of rapamycin in SA-induced protection (Figure 4). Many diseases were proved to be associated with autophagy, which provides clues for the treatment of diseases targeting autophagy. Our findings identified SA as a critical regulator of cerebral ischemia-induced neuronal autophagy and provided mechanistic insights into cerebral ischemia progression and other autophagy-related disease.

SA can significantly improve ischemic stroke, but the mechanism through which it regulates neuronal autophagy has yet to be determined. As for one of the clues, our results from Western blotting showed that SA inhibited the increase of phosphorylation of AMPK (Figure 5A and Figure 7E) and decrease of phosphorylation mTOR (Figure 5B and Figure 7F) induced by ischemic attack. AMPK is a stress-activated protein kinase that is activated when the cells sense an energy crisis. mTOR is a serine/threonine kinase, which is located downstream of AMPK and promotes anabolic metabolism and inhibits autophagy induction [42]. So far, the role of the AMPK/mTOR pathway in I/R-induced autophagy was documented in many studies. It suggested that the AMPK-autophagy pathway was activated, concomitant with mTOR inhibition in cerebral cortex after ischemic injury in mice. Moreover, inhibition of AMPK activity by Compound C inhibited autophagy and conferred protection against brain damage by restoring mTOR activity [43]. Puerarin, an isoflavonoid derived from *Radix puerariae*, could alleviate autophagy by inhibiting the APMK-mTOR-ULK1 signaling pathway, accompanied by reductions in infract volume and neurological deficits during ischemic stroke [30]. Similarly, PD149163 dramatically reduced JNK and AMPK/mTOR signaling pathway activation, and thereby inhibited autophagy to exert a neuroprotective effect [44]. These data suggested that autophagy inhibition by AMPK inactive was protective against cerebral ischemia. As expected, the AMPK inducer AICAR partially abolished the SA-induced protective effects and autophagy inhibition in our in vitro model (Figure 6).

Emerging data from preclinical studies and randomized control trials suggest that combination therapy provides survival advantages and increases the treatment effect for ischemic stroke without substantially increasing the side effects [45]. In this study, we demonstrated for the first time a potent synergistic effect of SA and 3-MA/Compound C on OGD/R-injured PC12 cells, which is mainly characterized by the inhibition of autophagy. The effects of the combined treatment of SA and 3-MA or Compound C on cell viability were better than that of SA monotherapy (70.5 ± 4.31% vs. 60.2 ± 7.65%, 76.5 ± 14.31% vs. 58.1 ± 6.95%), which showed synergistic therapeutic effect of compatibility. These findings provide some clues for the clinical application of SA and its combination with autophagy inhibitors or AMPK inhibitors.

## 4. Materials and Methods

### 4.1. Antibodies and Reagents

Antibodies against Beclin 1 and LC3B were obtained from Abcam (Cambridge, UK). Anti-AMPK, anti-mTOR, anti-p-AMPK, and anti-p-mTOR antibodies were purchased from Cell Signaling Technology (Danvers, MA, USA). The 3-MA was from Sigma-Aldrich (St. Louis, MO, USA). Rapamycin, Bafilomycin A1, AICAR, and Compound C were obtained from Selleck Chemicals (Houston, TX, USA). ExFect Transfection Reagent and TUNEL BrightGreen Apoptosis Detection Kit were purchased from Vazyme Biotech Co.,Ltd (Nanjing, China). The 3-(4, 5-dimethylthiazol-2-yl)-2 and 5-diphenyl tetrazolium bromide (MTT) were purchased from Amresco (Solon, OH, USA). Sch A (purity > 98%) was purchased from Aladdin (Shanghai, China).

### 4.2. Animals

Specified-pathogen-free (SPF) C57BL/6J mice (Male, 18–22 g) were purchased from the Reference Animal Research Centre of Yangzhou University (Yangzhou, China; certificate no SCXK 2017-0001). All experimental protocols were carried out according to the National Institutes of Health (NIH) guidelines, and the research was approved by the Institutional Animal Care and Use Committee of the Animal Ethics Committee of the School of Chinese Materia Medica, China, Pharmaceutical University.

### 4.3. Cell Culture

Highly differentiated PC12 cells were obtained from the Shanghai Institute of Cell Biology (Shanghai, China). The cells were cultured in DMEM, supplemented with 10% fetal bovine serum at 37 °C in a humidified cell culture incubator in 5% CO_2_/95% air.

### 4.4. OGD/R and Drug Treatments

OGD/R model was prepared in PC12 cells. SA, bafilomaycin, rapamycin, 3-MA, AICAR, and Compound C was dissolved in DMEM culture medium, without glucose, at various concentrations (SA: 10 µM, 3-MA: 3 mM, rapamycin: 0.1 μM, AICAR: 1 mM, Compound C: 1 µM, bafilomaycin: 100 nM) to adjust the final dimethyl sulfoxide (DMSO) concentration to 0.1% (*v*/*v*). After the cells were treated with drugs, OGD/R was induced in the cells for 6 h in a hypoxia chamber in DMEM culture medium without glucose, in an atmosphere of 5% CO_2_, 94% N_2_, and 1% O_2_, followed by culture under normoxic conditions.

### 4.5. TUNEL Staining

Apoptosis in PC12 cells subjected to various treatments was detected using TUNEL staining, which is a method to observe DNA strand breaks in nuclei. For TUNEL staining, PC12 cells were seeded on 35 mm confocal dishes (Glass Bottom Dish) at a density of 1 × 10^5^ cells/mL. After OGD/R, the immunofluorescence TUNEL assay was performed according to the instructions of the manufacturer. Nuclei were visualized with DAPI. Images were obtained by confocal microscopy confocal laser scanning microscope (Leica, LSM700, Mannheim, Germany).

### 4.6. Quantification of GFP-LC3 Puncta

Plasmids of LC3B (S8469-1A) were purchased from the FulenGen Company (Guangzhou, China). PC12 cells growing on 6-well plates at 50%–60% confluency were transfected with 4 µg of LC3B plasmid and 8 µL of ExFect Transfection Reagent in the Opti-MEM medium. The cells were incubated with the medium containing LC3B plasmid for 6 h. The transfection medium was then replaced with complete medium without antibiotics. After transfection for 48 h, cells were harvested for subsequent experiments.

### 4.7. Cell Viability

Cellular viability was evaluated using an MTT assay. Cells were seeded into 96-well culture plates at a density of 1 × 10^5^ cells/mL and were cultured for 24 h before further treatment. MTT (5 mg/mL) was added to each well and incubated at 37 °C for 4 h. After incubation, the medium was aspirated and 150 μL of DMSO was added to each well. The absorbance value with dual waves at 570 and 650 nm were read using a microplate reader (Epoch, Bio Tek, Winooski, VT, USA). Cell viability was expressed as a percentage with the control cells, which was taken as 100%.

### 4.8. In Vivo Cerebral Ischemia Model

Middle cerebral artery occlusion/reperfusion (MCAO/R) model was prepared in mice as follows: Briefly, cerebral ischemia was produced by intraluminal occlusion of the right middle cerebral artery, using a silicone rubber-coated 6-0 nylon monofilament. Meanwhile, sham-operations were carried out with the same procedure, except that the suture was not advanced into the internal carotid artery. In order to confirm the cerebral artery blood flow, a laser doppler flow meter (LDF; FLPI2, Moor, UK) was used. About 1 h after occlusion, the suture was withdrawn to allow reperfusion for 24 h. SA was injected immediately before reperfusion, 1 h after MCAO. The 40 mice were divided randomly into four groups (n = 10 in each group): sham operated (Sham), Sham + SA (40 mg/kg), I/R, and the I/R + SA groups (40 mg/kg) groups. SA was dissolved in saline solution containing 5% ethanol for injection, and the Sham group was administered vehicle.

### 4.9. In Vivo Immunofluorescence

After perfusion with PBS and 4% paraformaldehyde for 3 min, brain tissues were removed and placed into 4% paraformaldehyde at 4 °C. After 1 day, brain tissue was dehydrated using 40% sucrose for 5 days, embedded in OTC, and frozen at −70 °C. Brain tissues were sectioned into slices of 10 µm thickness using a cryotome (Leica, Mannheim, Germany) and then placed on adhesion microscope slides (Citoglas, China). Brain sections were fixed in 4% paraformaldehyde, permeabilized with 0.3% Triton X-100 in PBS, blocked with 5% normal donkey serum, and incubated overnight at 4 °C with specific primary antibodies against Beclin 1, LC3B, and NeuN. Then, sections were incubated with corresponding secondary antibodies at room temperature. Fluorescent images of ischemic penumbra were observed under confocal laser scanning microscopy.

### 4.10. Western Blot Analysis

Ischemic penumbra tissues and cells were lysed in RIPA buffer with protease inhibitor cocktail and used for Western blotting. Cell lysates (30 μg) were separated by 10% SDS-PAGE. The proteins were then transferred onto Polyvinylidene fluoride (PVDF) membranes. The membranes were blocked with 5% BSA and incubated overnight with primary antibodies against GAPDH (1:5000, mouse antibody), Beclin 1 (1:1000, rabbit antibody), LC3B (1:1000, rabbit antibody), AMPK (1:1000, rabbit antibody), mTOR (1:1000, rabbit antibody), p-AMPK (1:1000, rabbit antibody), and p-mTOR (1:1000, rabbit antibody), followed by horseradish peroxidase (HRP)-conjugated anti-rabbit or anti-mouse secondary antibody (dilution 1:10000). The image was then detected with ECL and photographed using the Gel Imaging System (BioRad, Hercules, CA, USA).

### 4.11. Statistical Analysis

All data were expressed as the means ± SD from at least three independent experiments. The data were analyzed by Student’s *t* test for two group comparisons or one-way analysis of variance (ANOVA), followed by Dunnett’s post hoc test for multiple comparisons, using Graph Pad Prism 6.0 (Graph Pad Software, La Jolla, CA, USA). Differences were considered significant with a P-value of less than 0.05.

## 5. Conclusions

**Figure 8:**
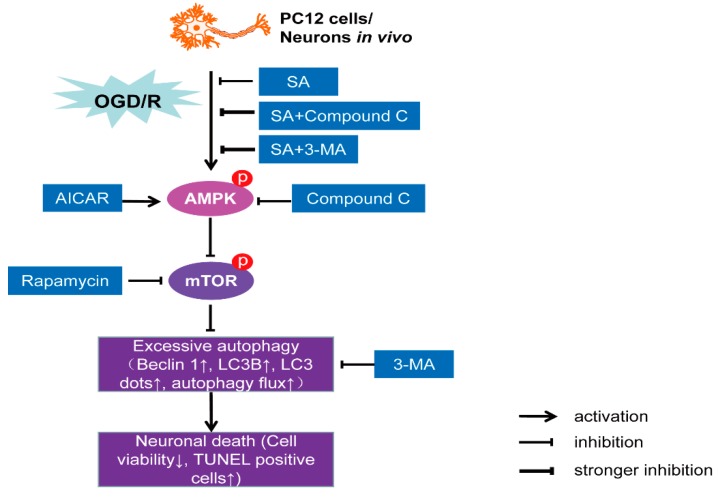
A proposed diagram tying together the observations involved in SA-induced neuroprotection against OGD/R injury. SA decreased phosphorylation of AMPK and increased phosphorylation of mTOR, which led to autophagy inhibition and neuroprotection against OGD/R injury in vivo and in vitro.

In conclusion, we propose an intriguing mechanism whereby SA protects against I/R-induced neuronal injury via inactivation of autophagy through the AMPK-mTOR pathway (Figure 8). These results provide a better understanding of the molecular mechanisms associated with the neuroprotective effects of SA and may provide new insight into a better design of neuroprotective agents against ischemic stroke.

## Supplementary Materials

The following are available online, Figure S1: LC3 expression in PC12 cells.
