# Supplementary material for: Schizandrin Protects against OGD/R-Induced Neuronal Injury by Suppressing Autophagy: Involvement of the AMPK/mTOR Pathway
Source: Molecules. 2019 Oct 8;24(19):3624. doi: 10.3390/molecules24193624 (PMC6804185; doi:10.3390/molecules24193624)
Supplement: Supplementary file 1 [file molecules-24-03624-s001.pdf]

Figure S1

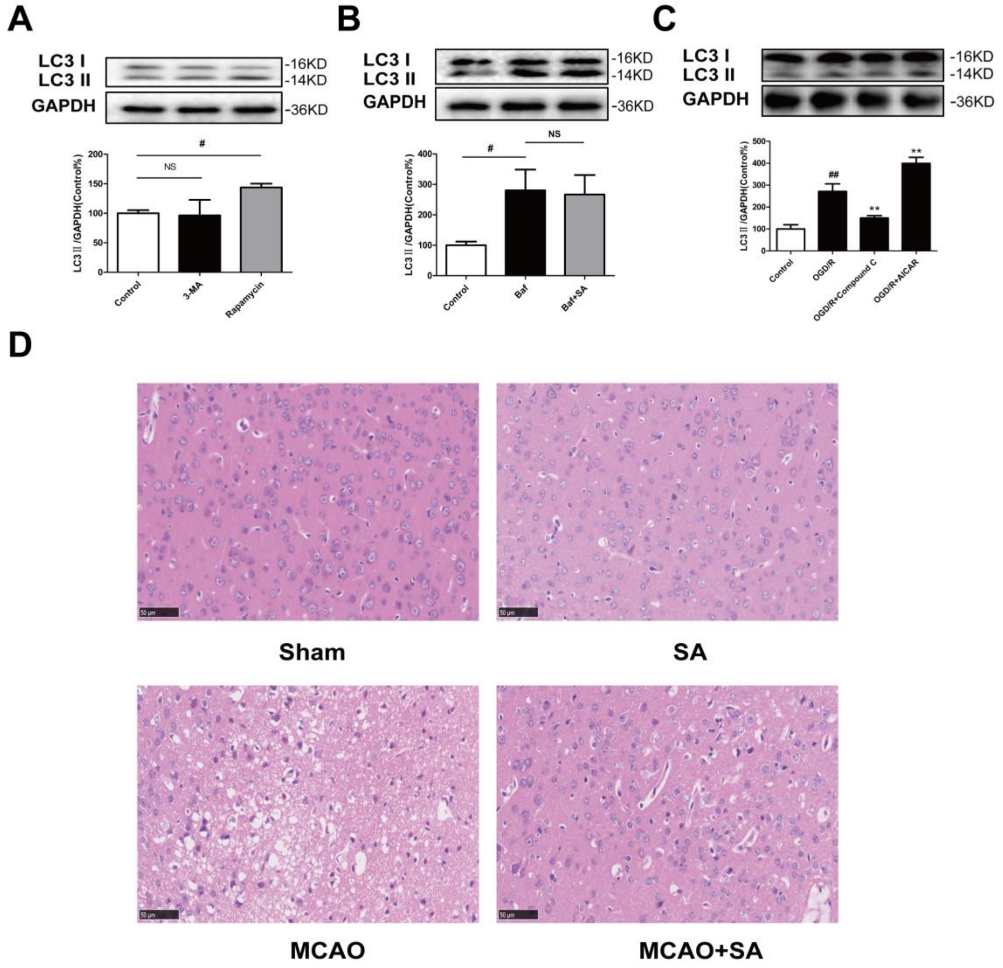

Figure S1. LC3 expression in PC12 cells. (A) PC12 cells were treated with rapamycin or 3-MA and then exposed to OGD/R; (B) PC12 cells were treated with baf or SA and then exposed to OGD/R; (C) PC12 cells were treated with compound C or AICAR and then exposed to OGD/R; Then the expressiona of LC3 were detected by western blot (n=3). (D) HE staining showing the morphological characteristics of mouse brains upon MCAO/R. Bar: 50 μm. All data are mean ±SD. $^{\#\#}P<0.01$ vs. control group; $^{*}P<0.05$ and $^{**}P<0.01$ vs. OGD/R group.
